# Validation data supporting the characterization of novel copper complexes as anticancer agents

**DOI:** 10.1016/j.dib.2016.11.063

**Published:** 2016-11-22

**Authors:** Ceyda Acilan, Buse Cevatemre, Zelal Adiguzel, Didem Karakas, Engin Ulukaya, Nádia Ribeiro, Isabel Correia, João Costa Pessoa

**Affiliations:** a TUBITAK, Marmara Research Center, Genetic Engineering and Biotechnology Institute, Gebze, Kocaeli, Turkey; b Uludag University, Faculty of Arts and Sciences, Department of Biology, Bursa, Turkey; c Uludag University, Medical School, Department of Medical Biochemistry, Bursa, Turkey; d Centro de Química Estrutural, Instituto Superior Técnico, Universidade de Lisboa, Av. Rovisco Pais 1, 1049-001 Lisbon, Portugal

## Abstract

Three copper(II) complexes, Cu(Sal-Gly)(phen), Cu(Sal-Gly)pheamine, Cu(Sal-Gly)phepoxy were synthesized and characterized for their anticancer properties and mechanism of action (Acilan et al., in press) [1]. Here, we provide supporting data on colon cancer cell lines complementing our previous findings in cervix cells. This paper also contains a data table for the fold changes and *p*-values of all genes analyzed in this study via a custom RT-qPCR array. All compounds induced DNA damage (based on 8-oxo-guanidine, ɣH2AX staining in cells) and apoptosis (based on elevated DNA condensation/fragmentation, Annexin V staining, caspase 3/7 activity and mitochondrial membrane depolarization) in HCT-116 colon cancer cells. The increase in oxidative stress was also further confirmed in these cells. Further interpretation of the data presented here can be found in the article entitled “Synthesis, biological characterization and evaluation of molecular mechanisms of novel copper complexes as anticancer agents” (Acilan et al., in press) [1].

**Specifications Table**

**Table:** 

Subject area	*Chemistry, Biology*
More specific subject area	*Copper(II) complexes, Molecular biology, Cancer biology, Drug development*
Type of data	*Table, microscopic image, graph, figure, scheme*
How data was acquired	*Microscopy (Leica DMI 6000), Circular dichroism (Jasco 720), Electron Paramagnetic Resonance (Bruker ESP 300E), UV–vis absorption (Perkin Elmer Lambda 35), Flow cytometry (Muse Cell Analyzer, EMD Millipore)*
Data format	*Analyzed*
Experimental factors	*None*
Experimental features	*Validation of the DNA binding properties of the presented Cu-complexes, apoptosis, oxidative stress and DNA damage induced by these complexes in human colon cancer cells*
Data source location	*Kocaeli/Turkey and Lisbon/Portugal*
Data accessibility	*Data are supplied with this article.*

**Value of the data**

• The data allows evaluating the stability of the Cu-complexes in aqueous buffers.
• The presented data displays evidence for the potential anticancer activity of Cu-complexes in human colon carcinoma HCT-116 cells. Therefore, the findings in our original article are not specific for only one cell line, but rather more general.
• The table may be valuable in determining/comparing potential molecular targets of other Cu-complexes.
• The data may give insight for researchers to design better therapeutic agents and offer a base for comparison between different compounds.

## 1 Data

**Scheme 1:**
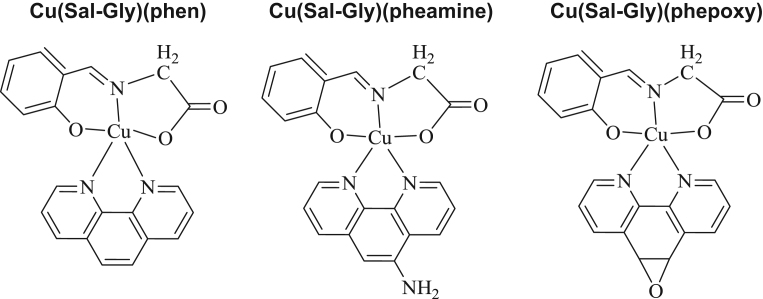
Formulation of the complexes.

There is still a great surge for potent anticancer agents with well-described activity. In today׳s world, most successful drugs find their place in the market among thousands of molecules, which were initially synthesized by the educated design of novel molecules with the aim of obtaining ameliorated properties. Therefore, data describing how different molecules act in different types of cells, become of great use. The desired function of anticancer drugs is inhibition of cell proliferation or survival, preferably with activity in cells with different genetic backgrounds, since every cancer is also divergent from one another. Here, we demonstrate the molecular mechanism of action of three different Cu-compounds (see Scheme 1) in HCT-116 cells supporting our previous findings with HeLa cells. We also disclose the full list of genes with fold change and exact *p*-values of our RT-qPCR analysis in response to Cu(Sal-Gly)(pheamine), the most specific compound against cancer cells (Table 1).

Most metallodrugs are not water soluble, and a small % or organic solvent is typically used in biological studies with metal complexes. On the other hand, biomolecules and cells are very sensitive to organic solvents. Therefore it is very important to check the stability of the complexes in aqueous environments; to make sure the compounds will maintain their structure for the necessary time period, without substantial degradation or precipitation prior to the biological evaluation, and to evaluate the effect of the organic solvent in the biological molecules under study, e.g. DNA.

**Fig. 1:**
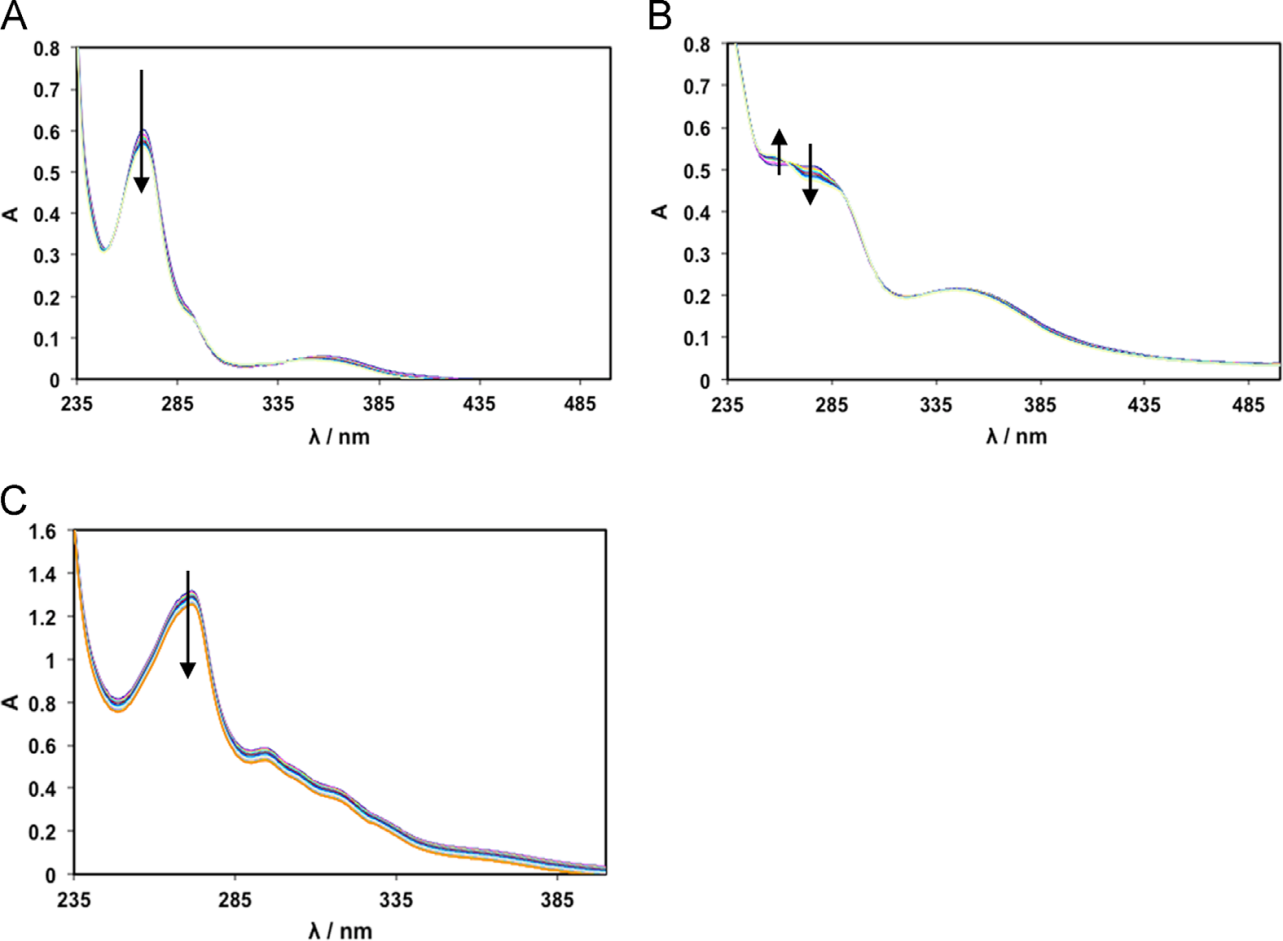
UV–vis absorption spectra measured with increasing time (time interval between spectra=5 min) for solutions of the Cu-complexes in PBS. (A) Cu(Sal-Gly)(phen) 20 μM (0.6% DMSO) total time=55 min; (B) [Cu(Sal-Gly)(pheamine) 20 μM (0.6% DMSO) total time=55 min and (C) [Cu(Sal-Gly)(phepoxy) 60 μM (1% DMSO) total time=100 min. Arrows indicate changes with time.

**Fig. 2:**
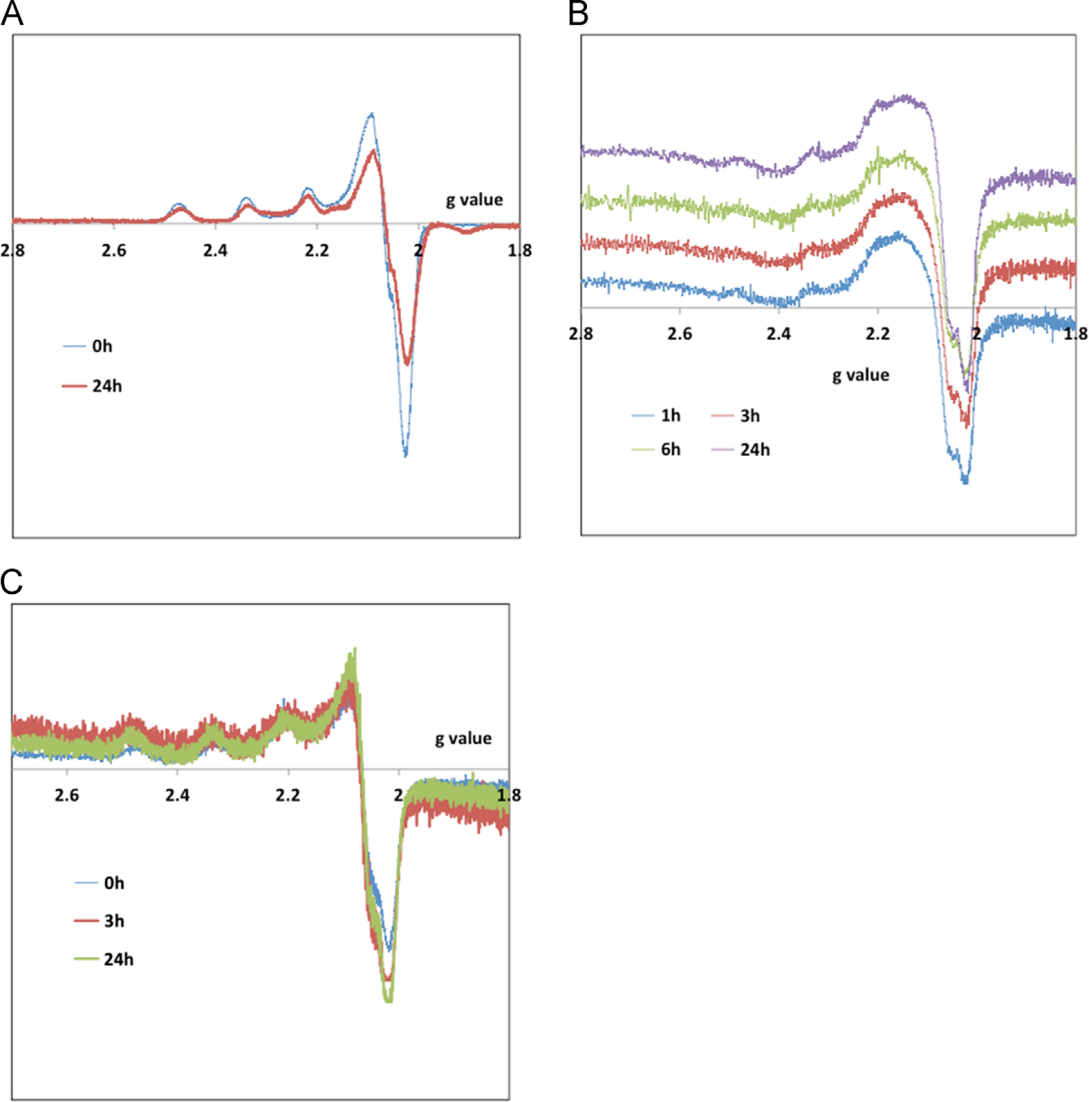
First derivative X-band EPR spectra measured for frozen solutions (77 K) of the complexes with time. (A) Cu(Sal-Gly)(phen) 4.0 mM in MeOH, and Cu(Sal-Gly)(pheamino) (B) and Cu(Sal-Gly)(phepoxy) 1.0 mM in DMSO.

**Fig. 3:**
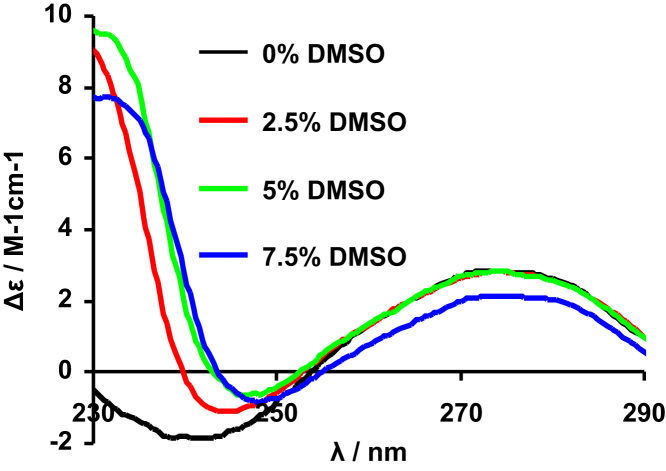
Circular dichroism spectra (1 cm optical path) of CT-DNA (60 μM) in the absence and presence of different % (v/v) of DMSO.

**Fig. 4:**
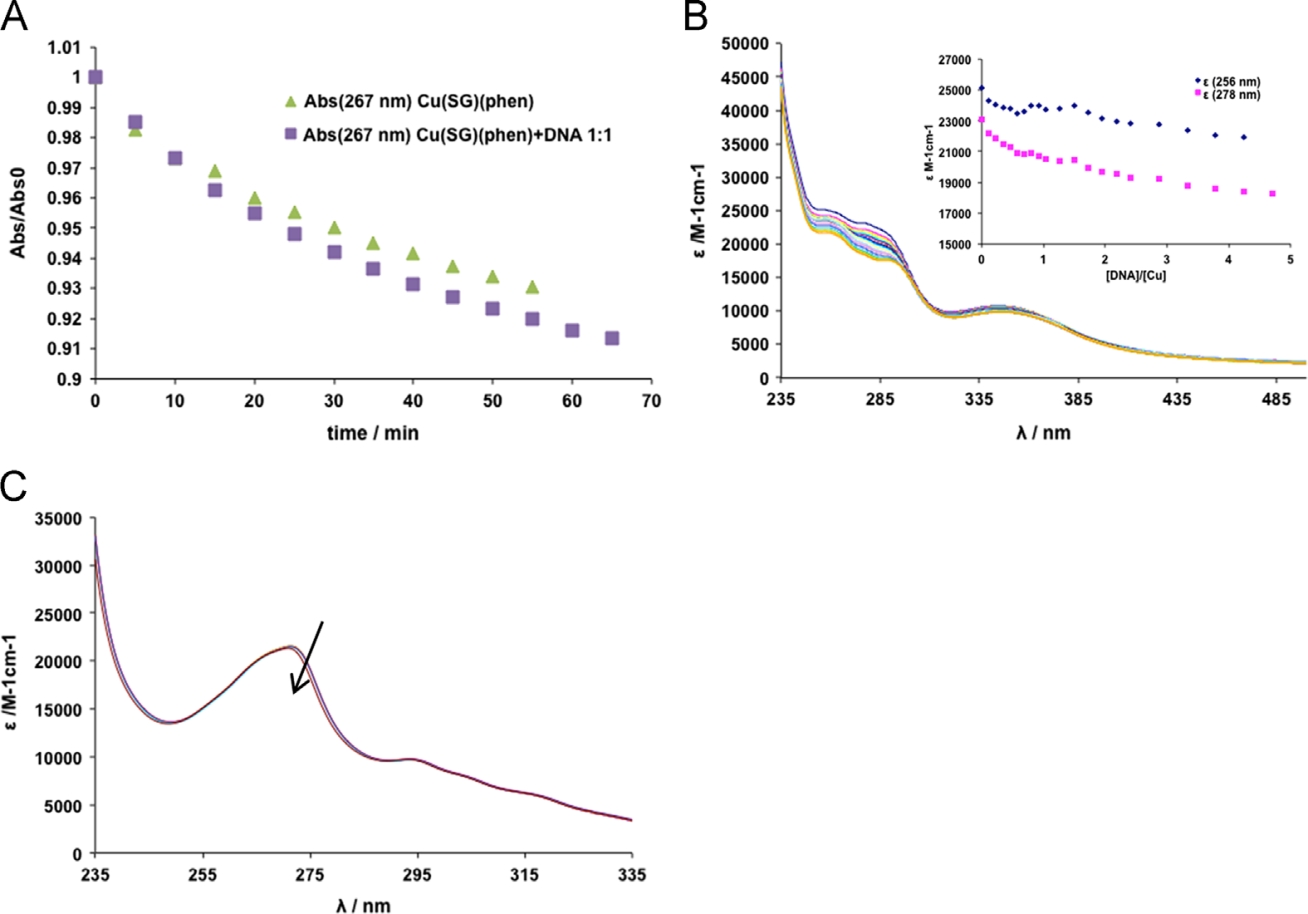
UV–vis absorption data: (A) Relative absorption values measured with time for solutions containing Cu(Sal-Gly)(phen) (20 μM) with and without CT-DNA (1 mol equivalent); (B) UV–vis absorption spectra measured for a solution of Cu(Sal-Gly)(pheamine) (40 μM) and increasing amounts of CT-DNA (from 0 to 180 μM); inset – changes observed in the *ε* values (M^−1^cm^−1^) at 256 and 278 nm. (C) UV–vis absorption spectra measured for a solution of Cu(Sal-Gly)(phepoxy) (27 μM) and increasing amounts of CT-DNA (from 0 to 28 μM). The arrow indicates increasing DNA concentration.

The complexes׳ stability in aqueous media (see Fig. 1) and organic solvents (Fig. 2) and was confirmed by measuring spectral changes within 1–2 h (UV–vis) and EPR (up to 24 h) after preparation of the solutions. Circular dichroism spectra of CT-DNA in the absence and presence of different % (v/v) of DMSO allowed evaluating the % range of DMSO where no changes in DNA configuration are detected (Fig. 3). The effect of adding DNA to the complexes׳ solutions was also evaluated by UV–vis spectroscopy (Fig. 4), which did not allow the determination of binding constants, but clearly showed occurring changes.

**Fig. 5:**
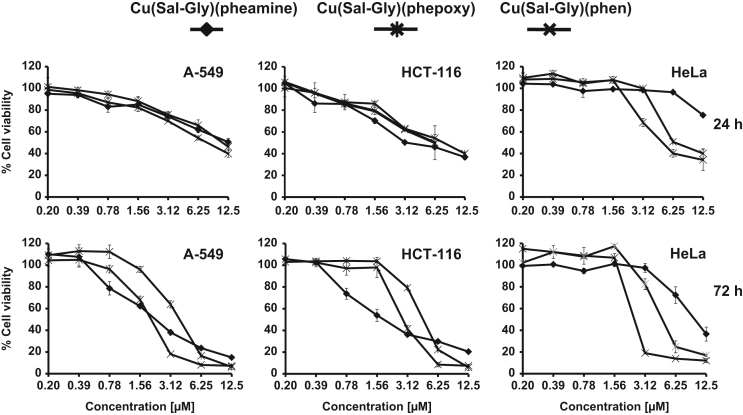
Cytotoxicity of the Cu compounds as determined by SRB analysis. Cytotoxicity in response to three different Cu-complexes was reevaluated using a different viability assay (SRB technique) upon increasing doses (0–12.5 µM) at different time points (24 h, 72 h) in a subset of cancer cells (A-549, HCT-116, HeLa). *x*-axis: concentration in µM, *y*-axis: cell viability normalized to untreated controls.

The cytotoxicity of the Cu-complexes were verified using a different assay relying on the amount of total proteins (Sulforhodamine B (SRB) Cell Viability Assay), as an alternative to measurement of change in mitochondrial dehydrogenase enzyme (MTT assay) in A-549, HCT-116 and HeLa cells (Fig. 5).

**Fig. 6:**
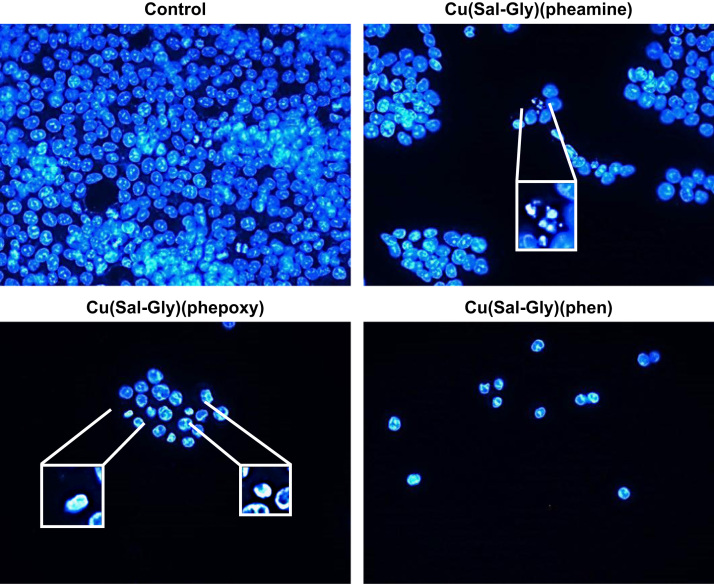
Changes in nuclear morphology in response to Cu complexes. HCT-116 cells displayed typical features of apoptosis such as fragmentation and condensation. HCT-116 cells treated with 12.5 μM of Cu complexes are shown in the figure. Insets indicate enlarged views of selected cells exhibiting these features.

**Fig. 7:**
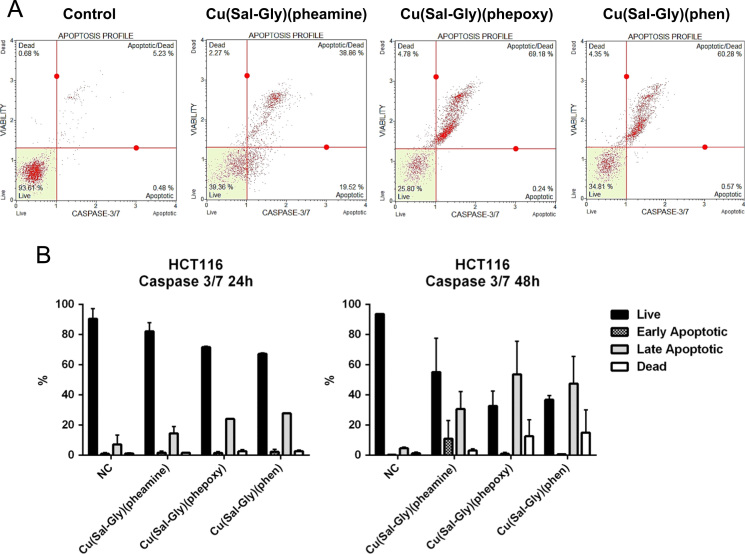
Annexin V/PI staining supports apoptotic form of cell death in response to Cu compounds. HCT-116 cells were treated with the Cu complexes and were stained with Annexin V/dead cell marker and counted with a flow cytometer as described in materials and methods. (A) Representative plots for HCT-116 cells following 24 h drug exposure are shown in the figure. (B) The graphs represent averages from 2 independent experiments from 24 h of exposure (left graph) and 48 h of exposure (right graph), where 10.000 cells were scored. *x*-axis: % cells, *y*-axis: name of the drug, NC: negative control, mock treated cells.

**Fig. 8:**
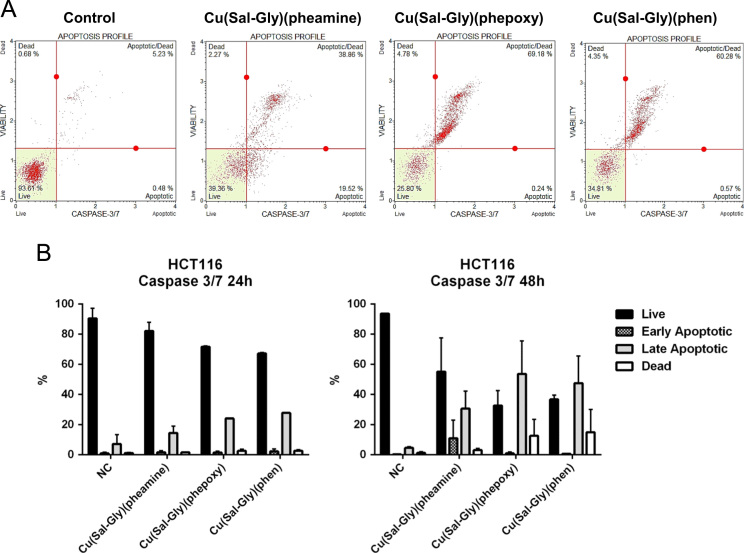
Analysis of caspase 3/7 activity using a flow cytometric assay. HCT-116 cells were treated with the Cu-complexes and were stained using Caspase 3/7 kit and counted with a flow cytometer as described in materials and methods. (A) Representative plots for HCT-116 cells following 48 h drug exposure are shown in the figure. (B) The graphs represent averages from 2 independent experiments from 24 h of exposure (left graph) and 48 h of exposure (right graph), where 10.000 cells were scored. *x*-axis: % cells, *y*-axis: name of the drug, NC: negative control, mock treated cells.

**Fig. 9:**
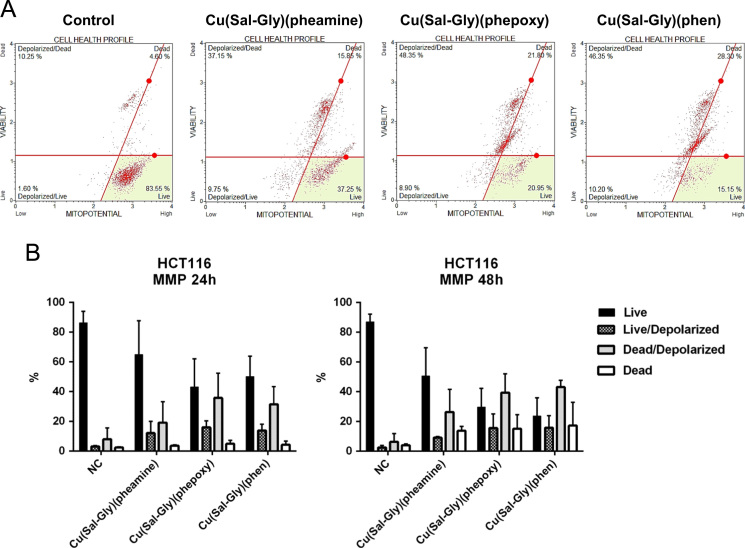
Induction of MMP in response to Cu complexes. HCT-116 cells were treated with the Cu complexes and were stained using MitoPotential kit and counted with a flow cytometer as described in materials and methods. (A) Representative plots for HCT-116 cells following 48 h drug exposure are shown in the figure. (B) The graphs represent averages from 2 independent experiments from 24 h of exposure (left graph) and 48 h of exposure (right graph), where 10.000 cells were scored. *x*-axis: % cells, *y*-axis: name of the drug, NC: negative control, mock treated cells.

Apoptosis was retested through the assessment of DNA condensation/fragmentation (Fig. 6), Annexin V staining (Fig. 7), caspase 3/7 activity (Fig. 8), and mitochondrial membrane depolarization (Fig. 9) in HCT-116 cells.

**Fig. 10:**
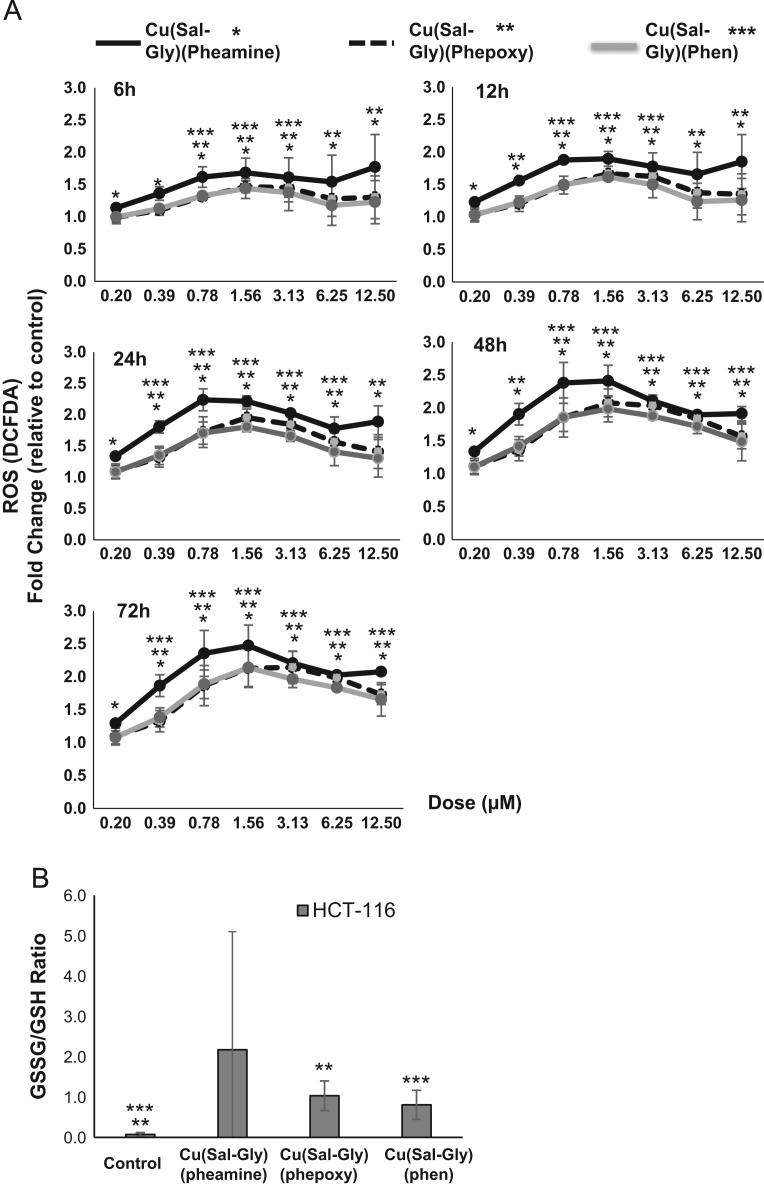
Increase in ROS in response to Cu-complexes. (A) Cells were pretreated with DCFDA with the indicated doses of Cu-complexes for 6–72 h and ROS were measured as described in materials and methods. Averages from three replicates from HCT-116 cells are shown in the graphs. *y*-axis: fold increase in DCFDA staining of cells relative to untreated controls, *x*-axis: concentration of Cu-complexes (µM). Asterisks indicate significance compared to untreated controls (paired samples *t*-test, *p*<0.05). (^⁎^:Cu(Sal-Gly)(pheamine), ^⁎⁎^: Cu(Sal-Gly)(phepoxy), ^⁎⁎⁎^: Cu(Sal-Gly)(phen)). (B) HCT-116 cells were treated with 12.5 µM of the Cu-complexes and lysed 24 h post incubation. The cellular GSSG/GSH (oxidized/reduced forms of glutathione) levels were measured, and an increase in oxidation was observed with all three Cu-complexes. Significance is indicated by asterisks (paired samples *t*-test, *p*<0.05).

The increase in oxidative stress was evaluated by the measurement of intracellular DCFDA (Fig. 10A) and the examination of oxidized glutathione (GSSG) by determining the ratio of GSSG/GSH (Fig. 10B) in HCT-116 cells.

**Fig. 11:**
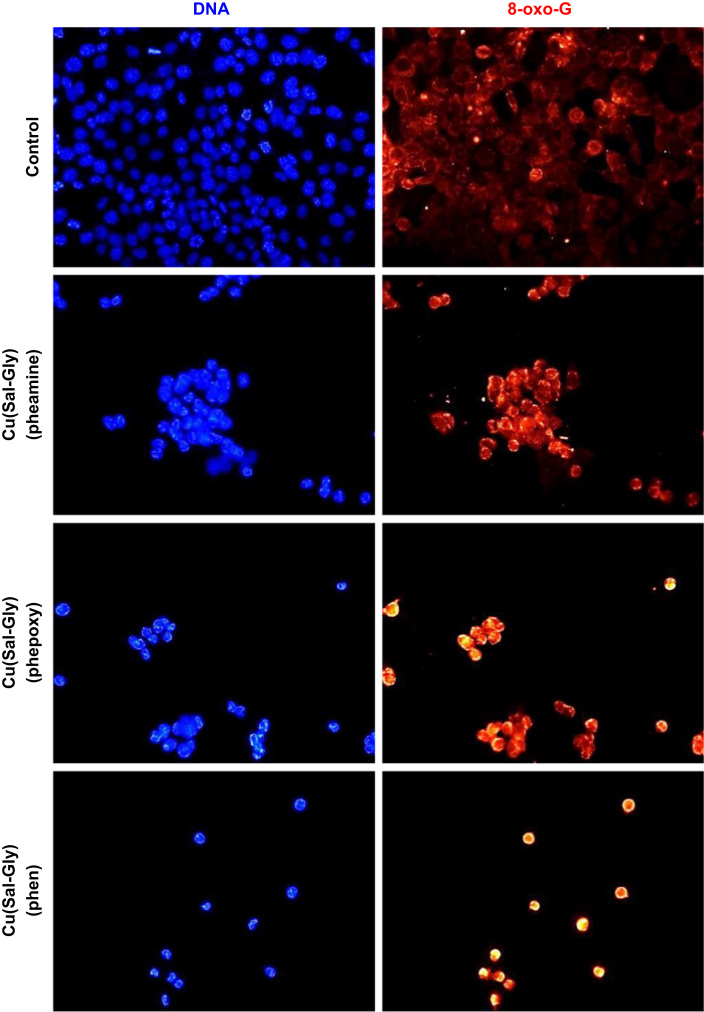
Oxidative DNA damage induced by the Cu-complexes. HCT-116 cells were treated with the 12.5 μM of Cu complexes for 24 h and were stained for DNA (blue) and 8-oxo-guanine (red), the most common lesion in response to oxidative stress. 8-oxo-G staining was increased upon treatment with all Cu complexes.

Consistent with our findings in HeLa cells, the compounds also appeared to induce oxidative DNA damage as assessed by 8-oxo-Guanindine staining, the most common lesion in DNA in response to oxidative stress (Fig. 11).

**Fig. 12:**
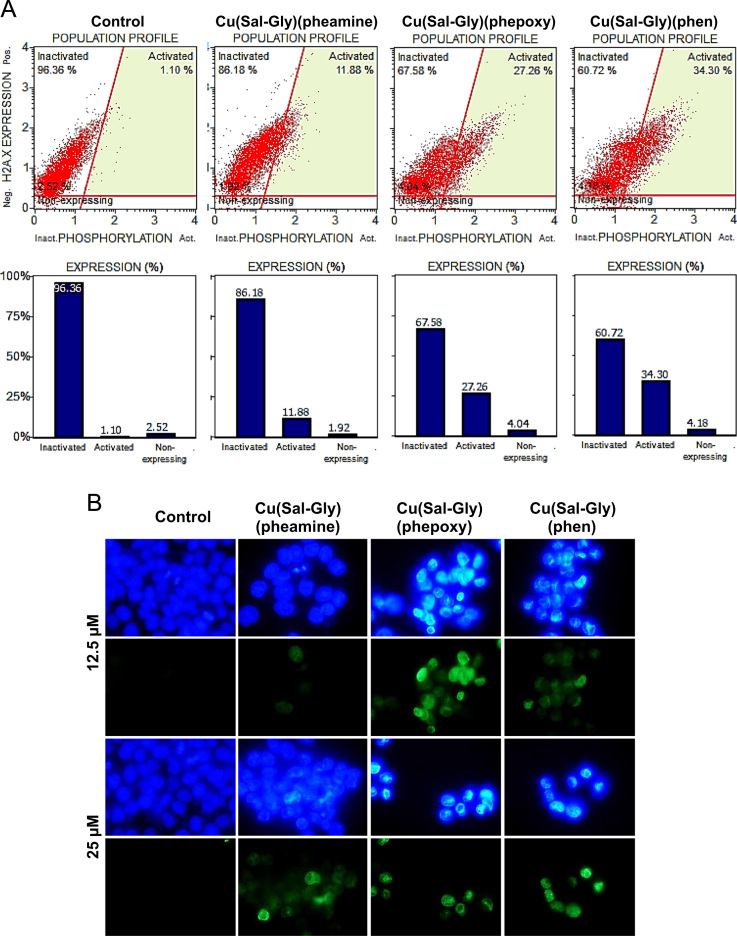
Induction DNA double strand breaks as a result of treatment with the Cu-complexes. (A) HCT-116 cells were treated with IC_90_ values of the Cu complexes for 12 h, and were stained with both anti-phospho-Histone H2AX (Ser139) and anti-Histone H2AX antibodies, and quantified using a flow cytometer. Non-expressing quadrant indicates cells that do not express H2AX antigen, inactivated quadrant indicates the cells expressing H2AX without phosphorylation and activated quadrant indicates the ɣH2AX phosphorylated cells. The quantification of results is shown in the graphs. (B) In order to visually determine ɣH2AX positivity, HCT-116 cells were treated with 12.5 and 25 µM of the Cu complexes, stained for ɣH2AX and observed under the fluorescence microscope. Images were taken using 100x magnification.

In addition to 8-oxo-Guanidine, there was an evident increase in double stranded DNA breaks as judged by ɣH2AX staining in HCT-116 cells both using flow cytometry (Fig. 12A) and microscopy (Fig. 12B).

## 2 Experimental design, materials and methods

### 2.1 Calf thymus DNA binding experiments

UV-Visible absorption (UV–vis) spectra were recorded on a Perkin-Elmer Lambda 35 spectrophotometer at room temperature. Circular dichroism (CD) spectra were recorded at 25 °C on a Jasco J-720 spectropolarimeter with an UV–vis (180–800 nm) photomultiplier (EXEL-308). EPR spectra were measured on a Bruker ESP 300E spectrometer at 77 K.

Millipore water was used for the preparation of TRIS and Phosphate Saline Buffer (PBS) buffers (0.10 M, pH=7.4). Calf thymus DNA (CT-DNA) was from Sigma (#D3664) and used as received. DNA stock solutions were prepared by dissolution in TRIS or PBS buffer. The stock solutions of the compounds were prepared by dissolving them in DMSO and dilution in TRIS buffer; they were used within a few hours.

Circular dichroism studies were done with ~3 mL solutions in quartz SUPRASIL^®^ cuvettes of 1 cm optical path. The CT-DNA solution was prepared by dilution of the stock solution in PBS buffer and its concentration (ca. 50–60 μM) was determined in each sample by measuring the absorbance at 260 nm, prior to addition of the Cu-complexes׳ stock solutions in DMSO (ca. 2.0–3.0 mM) to obtain each Cu:DNA ratio. UV–vis absorption titrations were done by adding aliquots of the DNA stock solution in PBS to solutions of the complexes (20 to 60 μM) in PBS (with 1% or less DMSO).

### 2.2 Cell culturing, drug treatment

All cancer cells (A-549, HCT-116, HeLa) were maintained in Dulbecco׳s Modified Eagle Medium/F12 (DMEM/F12, Sigma-Aldrich, #D0547) supplemented with fetal bovine serum (FBS, 5% for cancer cells and 10% for normal cells, Biochrom, #S0415) and penicillin/streptomycin (Biochrom, #A2212), and incubated at 37 °C, in 5% CO_2_. For viability assays, 5–8×10^3^ cells (~70–80% confluency depending on the cell line) were seeded in 96-well plates in regular culture medium, overnight. The following day, serial dilutions of complexes, Cu(Sal-Gly)(pheamine), Cu(Sal-Gly)(phepoxy) and Cu(Sal-Gly)(phen) (0.19–12.5 µM), were freshly prepared and added to the cells. After 24 or 72 h, cell viability was measured using MTT reagent (3-(4,5-dimethylthiazol-2-yl)-2,5-diphenyltetrazolium bromide) (Sigma, #M5655) as described before [2].

### 2.3 Sulforhodamine B (SRB) cell viability assay

For SRB assay, 5–8×10^3^ cells (~70–80% confluency depending on the cell line) were seeded in 96-well plates in regular culture medium in triplicate, overnight. The following day, serial dilutions of complexes, Cu(Sal-Gly)(pheamine), Cu(Sal-Gly)(phepoxy) and Cu(Sal-Gly)(phen) (0.19–12.5 µM), were freshly prepared and added to the cells. After 24 or 72 h, viable cells were fixed with the 50% trichloroacetic acid (TCA) at a final concentration of 10%. Plates were kept at 4 °C for 1 h, the supernatant was discarded and the plate was washed with deionized water five times. TCA-fixed cells were stained with SRB solution (0.4% in 1% acetic acid) for 30 min at room temperature (RT). Unbound SRB was removed by washing with 1% acetic acid and air-dried. Bound SRB stain was solubilized with Tris base solution (10 mM, pH:10.0), and plates were left on a shaker (10 min, 150 rpm). Absorbance was read by a spectrophotometer at 570 nm.

### 2.4 Detection of apoptosis

Apoptosis was detected though DNA condensation/fragmentation analysis using immunofluorescence staining (Leica DMI 6000 microscope) as described in [3]. Increase in Annexin V staining, caspase 3/7 activity and mitochondrial membrane depolarization were determined using the Muse Cell Analyzer (Millipore, Hayward, CA, USA) following manufacturer׳s protocols (Annexin V/Dead Cell kit MCH100105, Caspase 3/7 kit MCH100108, Muse MitoPotential kit MCH100110 respectively) and the details of the protocols are defined further in [1].

### 2.5 Detection of oxidative stress

Total reactive oxygen species were measured using using 2′,7′–dichlorofluorescein diacetate (DCFDA, Sigma, cat # D6883) following the protocol described in [1]. The cellular effects were determined through oxidation of Glutathione via measurement of GSSSG/GSH ratio (Promega, Madison, WI, USA, GSH/GSSG Glo assay kit) following manufacturer׳s protocols with a slight modification [1].

### 2.6 Immunofluorescence staining

For immunofluorescence, HCT-116 cells were fixed in freshly prepared 4% paraformaldehyde for 15 min at RT, permeabilized in 0.3% Triton X-100/PBS (1 h, RT), blocked in 0.2% gelatin (RT) and stained with ɣH2AX (Cell Signaling, #9718 S, 1:400) or 8-oxo-Guanine (EMD Millipore, MAB3560, 1:100) antibodies overnight at 4 °C.

### 2.7 ɣH2AX assay for the assessment of DNA damage using flow cytometry

HCT-116 cells were exposed to the Cu-complexes at the IC_90_ concentration for 12 h and stained using Muse ɣH2AX Activation Dual Detection (kit MCH200101, Millipore, Darmstadt, Germany) as described previously [1]. The data were acquired on the Muse Cell Analyzer (Millipore, Hayward, CA, USA).

## Figures and Tables

**Table 1:** List of genes studied in the RT-qPCR array. Three housekeeping genes were used in each experiment and each gene was normalized to the average of housekeeping genes. Fold change was calculated as the fold increase compared to untreated controls. An average of two independent experiments (each done in duplicate) is shown on the Table. Standard (Std) errors represent deviations from the mean, and the *p*-values were calculated using paired samples *t*-test using SPSS 17.0 software. Only one gene, Harakiri, was found to be statistically significant above the cut-off value of 1.5 fold.

**Gene name**	**Fold change**	**Std. deviation**	***p*-value**
Akt1	0.782	0.080	0.160
Akt2	1.033	0.142	0.797
APAF1	0.766	0.195	0.339
ATG7	1.029	0.036	0.465
ATM	0.841	0.103	0.274
ATR	0.968	0.199	0.858
AURKA	1.173	0.312	0.577
BAG1	1.048	0.028	0.243
BAG3	1.060	0.158	0.688
BAG4	1.163	0.386	0.657
Bak1	0.874	0.313	0.670
Bax	1.076	0.511	0.867
Bcl2	1.326	0.381	0.439
BCL2A1	ND	ND	ND
BCL10	0.778	0.315	0.501
BCL2L2	1.221	0.158	0.299
BCL2L10	1.309	0.841	0.695
BCLAF1	0.720	0.234	0.340
Bcl-xL	1.148	0.306	0.619
BECN1	1.312	0.322	0.402
BFAR	0.954	0.070	0.527
BIRC2	0.760	0.202	0.342
BIRC3	0.927	0.197	0.692
BIRC4	1.000	0.145	1.000
BIRC6	0.965	0.022	0.262
BIRC8	ND	ND	ND
Bid	1.091	0.323	0.758
Bik	0.926	0.451	0.856
Bim	1.203	0.332	0.546
BMI1	0.799	0.083	0.181
BNIP1	1.188	0.316	0.556
BNIP2	1.236	0.774	0.740
BNIP3	0.713	0.273	0.376
BNIP3L	0.745	0.261	0.398
CASP2	0.912	0.316	0.761
CASP3	0.695	0.199	0.274
CASP4	0.923	0.105	0.492
CASP5	1.903	2.615	0.711
CASP6	0.995	0.088	0.949
CASP7	0.763	0.062	0.117
CASP8	0.575	0.219	0.223
CASP9	0.757	0.576	0.657
CASP10	1.113	0.028	0.112
CASP14	2.676	2.644	0.535
CAT	1.082	0.178	0.633
CDC2	1.144	0.142	0.386
CDC25A	0.852	0.233	0.535
CDK2	0.817	0.109	0.251
CDK4	1.052	0.212	0.788
DCR	17.927	16.253	0.380
DR4	0.867	0.041	0.134
DR5	0.953	0.068	0.507
ERCC1	0.682	0.802	0.675
ERCC3	0.893	0.114	0.413
FADD	0.930	0.036	0.221
Fas(TNFRSF6)	0.837	0.065	0.175
GPX1	1.160	0.466	0.713
GRB2	0.897	0.060	0.250
GSTP1	1.121	0.333	0.697
HRK	1.731	0.327	0.000
LIG4	0.794	0.112	0.233
MCL1	0.709	0.334	0.434
MDM2	0.702	0.396	0.480
MUTYH	1.241	0.202	0.341
NFKB1/p50	0.793	0.050	0.107
Noxa	1.150	0.269	0.576
Nox1	2.129	0.989	0.353
Nox4	3.428	3.769	0.530
OGG1	1.121	0.104	0.349
PRDX1	0.964	0.155	0.800
Puma(BBC3)	1.348	0.517	0.516
P53	1.450	0.195	0.189
RAD51	0.895	0.151	0.503
RAD52	1.042	0.009	0.090
RIPK2	1.050	0.109	0.633
SIRT2	0.886	0.946	0.893
SOD1	0.942	0.185	0.732
TNFRSF11B(OPG)	2.180	1.581	0.483
XPA	1.116	0.079	0.286
XRCC5	0.862	0.005	0.016
